# SP-A binds alpha_1_-antitrypsin *in vitro *and reduces the association rate constant for neutrophil elastase

**DOI:** 10.1186/1465-9921-6-146

**Published:** 2005-12-13

**Authors:** Marina Gorrini, Anna Lupi, Paolo Iadarola, Conceição Dos Santos, Paola Rognoni, Daniele Dalzoppo, Natalia Carrabino, Ernesto Pozzi, Aldo Baritussio, Maurizio Luisetti

**Affiliations:** 1 Laboratorio di Biochimica e Genetica, Clinica di Malattie dell'Apparato Respiratorio, IRCCS Policlinico San Matteo, Università di Pavia, Pavia, Italy; 2 Clinica di Malattie dell'Apparato Respiratorio, IRCCS Policlinico San Matteo, Università di Pavia, Pavia, Italy; 3 Dipartimento di Biochimica "A. Castellani", Università di Pavia, Pavia, Italy; 4 Laboratorio Sperimentale di Ricerca Trapiantologia, Clinica Pediatrica, IRCCS Policlinico San Matteo, Università di Pavia, Pavia, Italy; 5 Istituto di Chimica Farmaceutica, Università di Padova, Padova, Italy; 6 Dipartimento di Scienze Mediche e Chirurgiche, Clinica Medica I, Università di Padova, Padova, Italy

## Abstract

**Background:**

α1-antitrypsin and surfactant protein-A (SP-A) are major lung defense proteins. With the hypothesis that SP-A could bind α1-antitrypsin, we designed a series of *in vitro *experiments aimed at investigating the nature and consequences of such an interaction.

**Methods and results:**

At an α1-antitrypsin:SP-A molar ratio of 1:1, the interaction resulted in a calcium-dependent decrease of 84.6% in the association rate constant of α1-antitrypsin for neutrophil elastase. The findings were similar when SP-A was coupled with the Z variant of α1-antitrypsin. The carbohydrate recognition domain of SP-A appeared to be a major determinant of the interaction, by recognizing α1-antitrypsin carbohydrate chains. However, binding of SP-A carbohydrate chains to the α1-antitrypsin amino acid backbone and interaction between carbohydrates of both proteins are also possible. Gel filtration chromatography and turnover per inactivation experiments indicated that one part of SP-A binds several molar parts of α1-antitrypsin.

**Conclusion:**

We conclude that the binding of SP-A to α1-antitrypsin results in a decrease of the inhibition of neutrophil elastase. This interaction could have potential implications in the physiologic regulation of α1-antitrypsin activity, in the pathogenesis of pulmonary emphysema, and in the defense against infectious agents.

## Background

Alpha_1_-antitrypsin (α_1_-AT) and surfactant protein-A (SP-A) are major defense glycoproteins in the alveolar spaces of human lungs. α_1_-AT, a 52,000 D glycoprotein, is secreted mostly by hepatocytes, and, to a lesser extent, by lung epithelial cells and phagocytes. α_1_-AT inhibits a variety of serine proteinases by its active site (Met358-Ser359), but its preferential target is human neutrophil elastase (HNE) as demonstrated by the high association rate constant (*K*_ass_) for this proteinase [1]. In the lungs, α_1_-AT protects the connective tissue from HNE released by triggered neutrophils; as a result, subjects homozygous for the common deficiency variant Z α_1_-AT (associated with 15% of normal plasma α_1_-AT levels) develop pulmonary emphysema early in life, especially if they smoke [2].

SP-A, a member of the *collectin *(*col*lagen-*lectin*) family [3], is one of the proteins of surfactant. Structurally, it comprises an N-terminal collagen-like domain connected by a neck to a C-terminal carbohydrate recognition domain (CRD) [4]. Six trimers are linked by disulfide bridges in an octadecamer of 650,000 D, in a "flower bouquet" alignment pattern [4,5]. A complex, predominantly triantennary, carbohydrate chain of ~4,000 D [6] is attached to the asparagine at position 187 of the CRD [7]. SP-A is mainly present in the alveoli in association with phospholipids, only 1% being present in the free form [8,9]. The primary function of surfactant is to reduce alveolar surface tension at end expiration. It is now however clear that SP-A, together with SP-D, another hydrophilic surfactant protein, plays a major role in the innate defenses of lung [5-10]. SP-A, in particular, is able to bind several micro-organisms and enhance their uptake by phagocytes, stimulate the production of free oxygen radicals, and induce phagocyte chemotaxis [11].

Most binding to micro-organisms, including influenza and herpes simplex viruses, Gram-positive and Gram-negative bacteria, mycobacteria, fungi, and *Pneumocystis carinii*, occurs via the CRD and is inhibited by sugars or calcium chelators [12].

Since some SP-A is present in the alveoli in the free form, it has a chance of coming into contact with α_1_-AT. We hypothesized that, in analogy with what happens with infectious agents, SP-A could bind to α_1_-AT, which carries 3 biantennary or triantennary asparagine-linked carbohydrate chains [13].

In this paper we provide *in vitro *evidence that the inhibitory activity of α_1_-AT towards HNE is significantly decreased in the presence of SP-A, probably as a consequence of SP-A binding to α_1_-AT. Such an interaction would represent a novel mechanism of regulating alveolar α_1_-AT. This could have relevance both for the pathogenesis of emphysema in patients with the Z α_1_-AT variant and for the lungs' defenses against infectious agents.

## Methods

### Preparative procedures

All reagents were of analytical grade, unless otherwise specified. The buffer used in all experiments was 0.2 M Na-K phosphate, with 0.5 M NaCl, 2 mM CaCl_2_, and 0.05% w/w Triton × 100, pH 8.0 (phosphate buffer), unless otherwise specified. Lipopolysaccharide (LPS) from *E. coli *serotype 026:B6 (Sigma) and methyl-α-D-mannopyranoside (MNOCH_3_) (Sigma) were dissolved in phosphate buffer. HNE and human α chymotrypsin (αChy) (ART, Athens, GA) were dissolved in 50 mM sodium acetate, 150 mM NaCl, pH 5.5 and diluted with phosphate buffer. N-glycocosidase F from *Flavobacterium meningosepticum *(PNGase F; EC 3.5.1.52) was purchased from Roche Diagnostics (Monza, Italy). *Clostridium histolyticum *collagenase type III (EC 3.4.24) came from Calbiochem (La Jolla, CA). The chromogenic substrates MeOSucAlaAlaProValNA (for HNE) and SucAlaAlaProPheNA (for αChy), and the irreversible inhibitors MeOSucAlaAlaProValCMK (for HNE) and TosPheCMK (for αChy) (all from Sigma) were dissolved in (CH_3_)_2_SO. Wild-type α_1_-antitryspin (M α_1_-AT) was either from ART or purified from human serum by covalent chromatography. Capillary isoelectric focusing (CIEF) with bare fused-silica capillaries filled with polyethylene oxide and carrier ampholyte solutions in the pH 3.5–5.0 range [14] was applied to confirm the presence of the common M α_1_-AT variant. Z α_1_-AT variant was purified by covalent chromatography from subjects identified within the Italian screening program for α_1_-AT deficiency [15]. SP-A was isolated as described [16] from surfactant obtained from 3 patients affected by pulmonary alveolar proteinosis (PAP), subjected to therapeutic whole lung lavage [17] and from adult New Zealand rabbits. To isolate surfactant, the bronchoalveolar lavage fluid was filtered through gauze and centrifuged at 150 g for 10 minutes. The supernatant was centrifuged for 30 minutes at 80,000 × g and the resulting pellet was suspended in 10 mM Tris-HCl pH 7.4, 145 mM NaCl, 1.25 mM CaCl_2_, 1 mM MgCl_2, _2.2 M sucrose (solution A), overlaid with 10 mM Tris-HCl pH 7.4, 145 mM NaCl, 1.25 mM CaCl_2_, 1 mM MgCl_2_, 2 M sucrose (solution B) and ultracentrifuged overnight at 85,000 × g in a Ti 60 rotor (Beckman). The floating material was dispersed in water and centrifuged for 30 minutes at 100,000 × g and the pellet recovered was stored at -70°C (purified surfactant). To obtain SP-A, surfactant was injected into a 50-fold excess by volume of 1-butanol and stirred at room temperature for 30 minutes. After centrifugation, the pellet was suspended in 1-butanol and re-centrifuged at 4,000 × g for 1 hour at room temperature. The final precipitate was dried under nitrogen and then resuspended in 5 mM Tris-HCl, 145 mM NaCl, 20 mM octyl β-D-glucopyranoside, pH 7.4 (solution C). After centrifugation at 100,000 × g for 1 hour, the pellet was resuspended in 5 mM Tris-HCl pH 7.4 (solution D) and dialyzed against solution D for 48 hours with at least six changes. The final solution was centrifuged at 100,000 × g for 1 hour and the resulting supernatant, containing purified SP-A, was stored. Endotoxin-free SP-A was obtained by treatment with polymyxin-B (Sigma). Small aliquots of SP-A in solution D were incubated in a 1:1 ratio for 6 hours at 4°C with polymyxin-agarose previously equilibrated with 5 mM Tris-HCl, 100 mM octyl β-D-glucopyranoside and 2 mM EDTA, pH 7.4. Polymyxin-agarose was removed by centrifugation at 14,000 × g for 15 minutes, and the supernatant was then dialyzed against 5 mM Tris-HCl pH 7.4 for 48 hours with at least six changes and lyophilized [17,18]. For some experiments polymyxin-treated SP-A was further purified by D-mannose sepharose 4B chromatography. SP-A was added to a small column containing D-mannose sepharose 4B (Pharmacia) previously equilibrated with 5 mM HEPES, 0.4% Triton × 100 and 1.5 mM CaCl_2_, pH 7.2 (solution E), and the column was washed extensively with solution E. SP-A was finally eluted with 5 mM HEPES, 0.4% Triton × 100 and 2.5 mM EDTA, pH 7.2 (solution F).

### Modification of the native proteins

**Figure 1:**
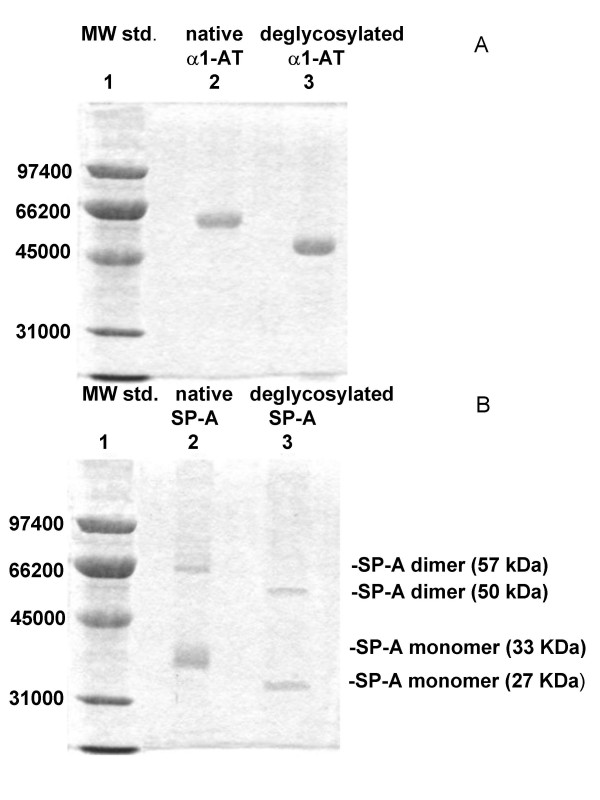
**SDS-PAGE under reducing conditions**. **A**: α_1_-AT; **B**: SP-A. *Lane 1*: molecular weights; *lane 2*: native protein; *lane 3*: deglycosylated protein. The two bands in *gel B, lanes 2 and 3 *correspond to dimers (57 and 50 kDa, respectively) and monomers (33 and 27 kDa, respectively) of SP-A.

Native and modified proteins used in our experiments were at high degree of purification (Figure 1). *See *additional file 1*for more details*.

### Identification of the SP-A/ α_1_-AT complex

#### 1) Gel filtration HPLC

A mixture of SP-A (1.62 mg/ml) and α_1_-AT (1 mg/ml) in a 1:50 molar ratio was incubated for 24 hrs at 37°C in phosphate buffer. The SP-A/α_1_-AT mixture and single proteins were loaded in a Jasco PU 980 HPLC system (Japan Spectroscopic, Tokyo, Japan) equipped with two Biosep-SEC-S 4000 columns (300 × 7.80 mm each, Phenomenex, Torrence, CA, USA) connected in series. Samples were eluted with 100 mM Na_2_HPO_4, _2 mM CaCl_2_, pH 6.8 at a flow rate of 0.3 ml/ min, and monitored at 220 nm. The excluded (V_0 _= 12.43 ml) and total (V_t _= 24.82 ml) volumes were determined using dextran and creatinine, respectively; a calibration curve was obtained by running through the column a set of standard proteins: α_2_-macroglobulin (725 kD), aldolase (158 kD), bovine serum albumin (67 kD), chymotrypsinogen (25 kD), and cytochrome C (12.5 kD). The results were reported as mean ± SD of three separate experiments. 2

#### 2) Qualitative immunodetection by ELISA

250 ng of standard α_1_-AT, purified SP-A, and SP-A/α_1_-AT complex collected from the Size Exclusion Chromatography experiments, were immobilized in 50 mM Na_2_CO_3_, pH 9.5 overnight at 4°C in a polypropylene plate (Corning, New York, USA). Plates were then brought at room temperature, washed with 150 mM NaCl, 0.1% Tween 20 (ELISA buffer), blocked for 1 h with 50 mM Na_2_CO_3_, 2% BSA pH 9.5, incubated for 2 hrs in the presence of primary antibodies diluted 1:500 (goat anti-human α_1_-AT and rabbit anti-human SP-A; ICN, Aurora, OH, USA), washed and finally reacted for 2 hrs with the appropriate biotinylated secondary antibodies diluted 1:5000 (Chemicon, Temecula, CA, USA). After washing, 100 μL of avidin diluted 1:2000 were added, and samples were incubated for 30 min. Color development was achieved by incubating the samples with 1,2-phenylenediamine dihydrochloride (Dako, Bucks, UK). The reaction was stopped by addition of 100 μl of 0.5 M H_2_SO_4 _and OD was read at 490 nm with a Bio-Rad 680 Microplate Reader (Bio-Rad Laboratories, CA, USA).

### Kinetics studies

Rate constants were derived by competition experiments of HNE and αChy. Kinetic parameters were determined as described [20,21]. The active sites of HNE and αChy were titrated using a procedure based on the measurement of pNa released after enzymatic cleavage of MeOSucAlaAlaProValNA and SucAlaAlaProPheNA, respectively, at 37°C [22]. Product formation was monitored spectrophotometrically at a wavelength of 405 nm using a Bio Rad Microplate Reader model 3550. To titrate the different forms of α_1_-ATs (α_1_-AT, deglycosylated α_1_-AT and Z α_1_-AT), 7.5 nM HNE was incubated for 15 min at 37°C with 0–100 nM inhibitor, in the presence of 2 mM MeOSucAlaAlaProValNA. All following kinetic experiments were derived from α_1_-ATs and SP-A/α_1_-ATs complexes (obtained by incubation of α_1_-ATs, from 0 to 25 nM, with SP-A 15, 7.5, 1.5, 0.15 mM for 15 min at 37°C). *See *additional file 1*for more details*.

## Results

To investigate the interaction between SP-A and α_1_-AT we studied whether *K*_ass _values, derived from incubating HNE with α_1_-AT, were modified by SP-A. Indeed we found a progressive decrease in the *K*_ass _as the SP-A concentrations increased (Table 1), irrespective of the animal source of SP-A. To exclude that the observed effect was due to LPS co-purified with SP-A [23], we repeated the assay using endotoxin-free SPA, but found no differences with native SP-A (Table 1). To reinforce this finding, in separate experiments we spiked α_1_-AT and SP-A/α_1_-AT mixtures with increasing amounts of LPS, without measurable effect on the *K*_ass _of α_1_-AT or SP-A/α_1_-AT mixture (not shown). As expected, [24], we found that the *K*_ass _of Z α_1_-AT for HNE was 3.5 fold lower than that of the normal, M α_1_-AT. When Z α_1_-AT was coupled with increasing SP-A concentrations, a further decrease in *K*_ass _towards HNE was observed (Table 2).

**Table 1:** Association rate constant (*K*_ass _M^-1^sec^-1^) for inhibition of HNE by α_1_-AT with SP-A. SP-A employed was both from humans affected by PAP or from rabbit, polymyxin treated and polymyxin-mannose treated. Data are means ± SD of three different experiments.

Reaction conditions		*K*_ass_, (M^-1^sec^-1^) means ± SD			
		Human SP-A			Rabbit SP-A

α_1_-AT nM	SP-A nM	*Native*	*Polymyxin-treated*	*Polymyxin/Mannose-treated*	*Native*

7.5	0	3.40 ± 0.0079 × 10^7^	3.40 ± 0.0079 × 10^7^	3.40 ± 0.0079 × 10^7^	3.40 ± 0.0079 × 10^7^
7.5	0.15	1.84 ± 0.0577 × 10^7^	1.84 ± 0.0580 × 10^7^	1.86 ± 0.0565 × 10^7^	1.82 ± 0.0585 × 10^7^
7.5	1.5	1.70 ± 0.0623 × 10^7^	1.70 ± 0.0631 × 10^7^	1.72 ± 0.0618 × 10^7^	1.68 ± 0.0620 × 10^7^
7.5	7.5	5.20 ± 0.0483 × 10^6^	5.22 ± 0.0480 × 10^6^	5.00 ± 0.0478 × 10^6^	5.40 ± 0.0490 × 10^6^
7.5	15	4.30 ± 0.0513 × 10^6^	4.30 ± 0.0520 × 10^6^	4.30 ± 0.0520 × 10^6^	4.40 ± 0.0498 × 10^6^

**Table 2:** Association rate constant for inhibition of HNE by α_1_-AT and Z α_1_-AT with SP-A. Data are means ± SD of experiments performed in triplicate with 3 different batches of human SP-A and1batch of rabbit SP-A.

α_1_-AT/SPA ratio	α_1_-AT				Z α_1_-AT			
	
	Reaction condition		*K*_ass_, (M^-1^sec^-1^)	decrease in *K*_ass_, n-fold	Reaction condition		*K*_ass_, (M^-1^sec^-1^)	decrease in *K*_ass_, n-fold
						
	α_1_-AT nM	SP-A nM			Z α_1_-AT nM	SP-A nM		
	7.5	0	3.40 ± 0.0079 × 10^7^	0	7.5	0	9.80 ± 0.0032 × 10^6^	0
50	7.5	0.15	1.84 ± 0.0577 × 10^7^	1.8	7.5	0.15	5.20 ± 0.0314 × 10^6^	1.9
5	7.5	1.5	1.70 ± 0.0623 × 10^7^	2	7.5	1.5	4.70 ± 0.0268 × 10^6^	2.1
1	7.5	7.5	5.20 ± 0.0483 × 10^6^	6.5	7.5	7.5	4.30 ± 0.0240 × 10^6^	2.3
0.5	7.5	15	4.30 ± 0.0513 × 10^6^	7.9	7.5	15	3.80 ± 0.0221 × 10^6^	2.6

To exclude that the results were due to non-specific binding, we incubated 7.5 nM HNE with 0–100 nM α_1_-AT for 15 min at 37°C in microtiter plates or in glass tubes and then measured the residual HNE activity with 2 mM MeOSucAlaAlaProValNA, finding no difference between plastics and glass. Furthermore, to exclude binding of SP-A to plastics we incubated 15 nM SP-A with I^125^α1-AT (from 0 to 100 nM) at 37°C. The number of Cpm of the samples with SP-A were the same of wells without proteins. We concluded that our data were compatible with binding of α_1_-AT to SP-A.

**Figure 2:**
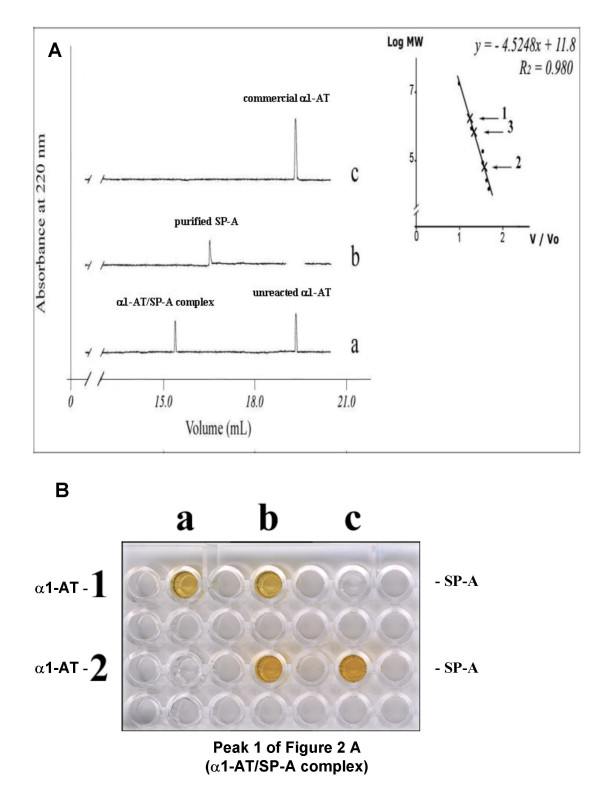
**Isolation and immunodetection of the α_1_-AT /SP-A complex**. **A**: Isolation of the complex by gel filtration chromatography on two Biosep SEC – S 4000 columns connected in series using HPLC. Gel filtration profiles: commercial α_1_-AT (*in profile c*; 19.32 ± 0.1 mL); purified SP-A (*in profile b*; 16.49 ± 0.07 mL); α_1_-AT /SP-A complex (*in profile a*; 15.31 ± 0.04 mL) and unreacted α_1_-AT (*in profile a*; 19.35 ± 0.09 mL). *Inset*: calibration curve obtained using the following standards: A = α_2_-macroglobulin (725 kDa), B = aldolase (158 kDa), C = bovine serum albumin (67 kDa), D = chymotrypsinogen (25 kDa), E = cytocrome C (12.5 kDa). **B**: Immunodetection of the complex. α_1_-AT was added to wells a1 and a2, peak 1 (α_1_-AT /SP-A complex) of Figure 2A was added to wells b1 and b2, and SP-A to wells c1 and c2. Antiserum anti-α_1_-AT was added to wells a1, b1 and c1, antiserum anti-SP-A was added to wells a2, b2 and c2. Peak 1 (α_1_-AT /SP-A complex) is recognized by both antisera.

Gel filtration HPLC was then used to determine the molecular weight of the SP-A/α_1_-AT complex. As shown in Figure 2A, profile a, a mixture of SP-A and α_1_-AT (1 mg/ml), gave two peaks, one corresponding to free _1_-AT (unreacted α_1_-AT) and one, with a theoretical molecular weight of 1,642 kD (_1_-AT/SP-A complex), possibly corresponding to a complex made by one molecule of SP-A (670 kD) and 18 molecules of α_1_-AT (54 kD), suggesting that, under the experimental conditions applied, each monomer of SP-A bound one molecule of α_1_-AT. Further evidence that the first peak of profile a (Figure 2A) contained the complex SP-A/α_1_-AT was obtained by using an immunochemical assay in which a polypropylene plate was probed with antisera anti α_1_-AT and anti SP-A. As shown in Figure 2B, the first peak in profile a of Figure 2A contained both α_1_-AT and SP-A.

**Figure 3:**
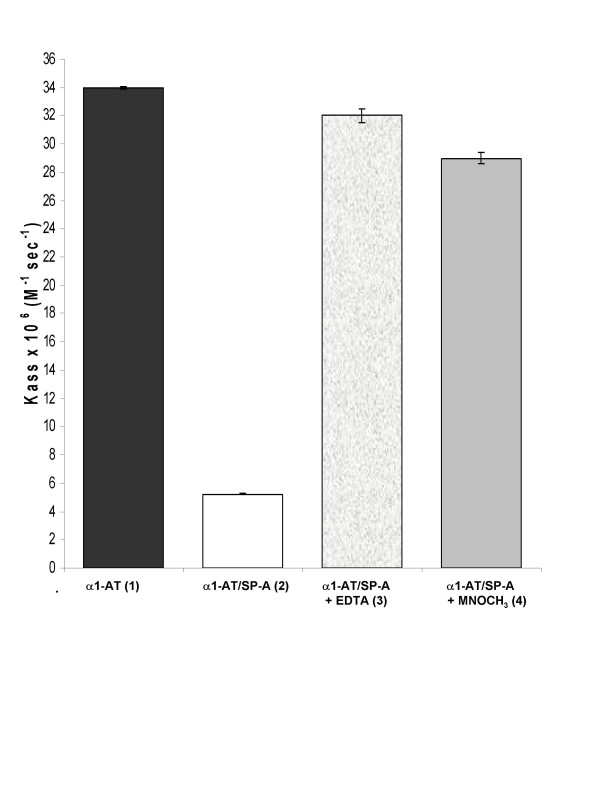
**Effects of calcium removal and sugar addition on K_ass _M^-1^sec^-1^**. Inhibition of HNE by α_1_-AT (7.5 nM), alone or coupled with 7.5 nM SP-A: (1) α_1_-AT alone, (2) α_1_-AT plus SP-A, (3) α_1_-AT plus SP-A with 5 mM EDTA and (4) α_1_-AT plus SP-A with 1 M MNOCH_3_. (Data are means ± SD of experiments performed in triplicate).

The effect of SP-A on the *K*_ass _of α_1_-AT for HNE was calcium-dependent, being abrogated by EDTA (Figure 3). Since the calcium-binding domain of SP-A lays at the COOH terminus, next to the CRD [25], we supposed that this part of SP-A could be involved in the binding of SP-A to α_1_-AT, *via *the α_1_-AT carbohydrate chains. Consistent with these findings, the addition of 1 M mannopyranoside to the SP-A/α_1_-AT mixture almost totally reversed the reduction in the *K*_ass _(Figure 3), most likely by interfering with the binding of CRD to α_1_-AT carbohydrate chains [26,27]. The fact that the lipid recognition domain of SP-A is located in the neck region of the molecule, far from the CRD [23], could explain the lack of influence of LPS on the binding of SP-A to α_1_-AT (Table 1).

**Figure 4:**
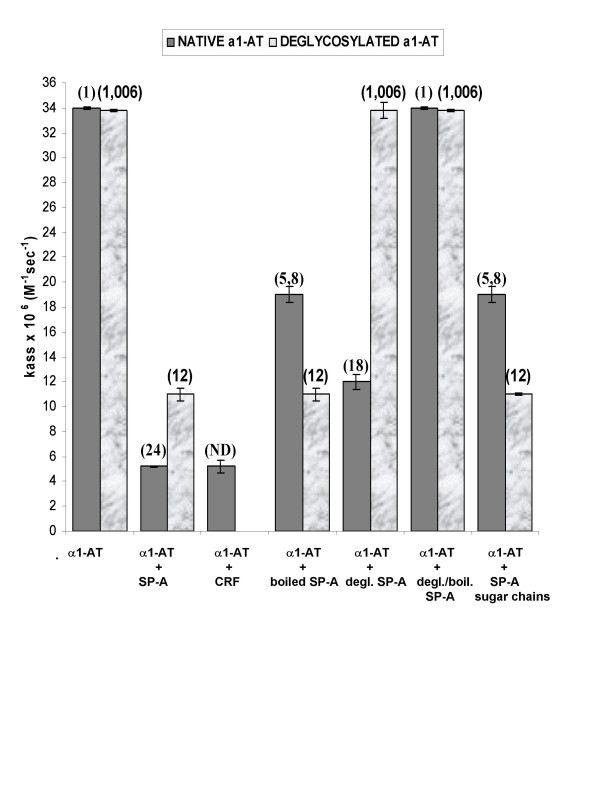
***K*_ass _M^-1^sec^-1 ^for inhibition of HNE by modified proteins (7.5 nM), alone or in combination**. *K*_ass _data are means ± SD of experiments performed in triplicate. SI values of the associations in bold at the top of the figure.

To better clarify the role of the CRD in the binding of SP-A to α_1_-AT, we modified both proteins by enzymatic digestion, deglycosylation or boiling and then used them to calculate the *K*_ass _of α_1_-AT for HNE and to deduce the molar parts of α_1_-AT bound to SP-A from the number of turnovers per inactivation of α_1_-AT not bound to SP-A. Thus we found that the CRD of SP-A appears to contain all the putative SP-A binding sites for α_1_-AT since, when incubated with α_1_-AT, it retained the same *K*_ass_, as that of native SP-A (Figure 4).

**Figure 5:**
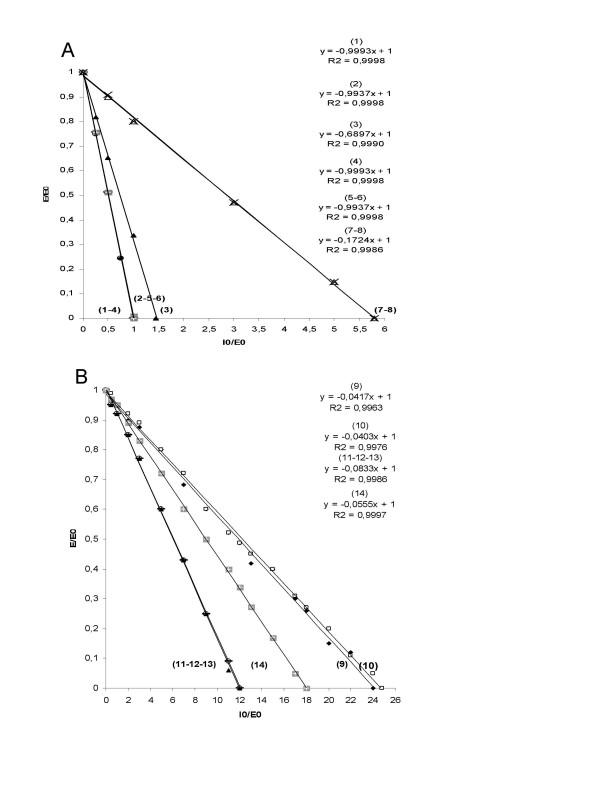
**Turnover numbers per inactivation**. Turnover numbers were determined plotting residual enzyme activity/initial enzyme activity versus initial inhibitor concentration/initial enzyme activity. **A**: (1) native α_1_-AT, (2) deglycosylated α_1_-AT, (3) Z α_1_-AT, (4) native α_1_-AT coupled with deglycosylated and boiled SP-A, (5) deglycosylated α_1_-AT coupled with deglycosylated SP-A, (6) deglycosylated α_1_-AT coupled with deglycosylated and boiled SP-A, (7) native α_1_-AT coupled with boiled SP-A and (8)native α_1_-AT coupled with SP-A sugar chains. **B**: (9) native α_1_-AT coupled with native SP-A and (10) Z α_1_-AT coupled with native SP-A, (11) deglycosylated α_1_-AT coupled with native SP-A, (12) deglycosylated α_1_-AT coupled with boiled SP-A, (13) deglycosylated α_1_-AT coupled with SP-A sugar chains and (14) native α_1_-AT coupled with deglycosylated SP-A.

Turnover per inactivation (also referred to as stoichiometry of inhibition (SI) or partition ratio + 1) defines the number of moles of irreversible inhibitor required to completely inhibit 1 mole of target proteinase. The turnover number resulting from the interaction between unmodified SP-A and α_1_-AT was 24, i.e. one part of SP-A binds 23 molar parts of α_1_-AT and 24 SP-A plus α_1_-AT binds inhibit 1 part of enzyme (Figure 5). The same binding pattern emerged when Z α_1_-AT was used instead of α_1_-AT, suggesting that the difference in the *K*_ass _between the two variants of α_1_-AT is independent of the number of molar parts of inhibitor bound to SP-A.

Deglycosylated α_1_-AT retains its ability to inhibit HNE (*K*_ass _3.38 × 10^7 ^M^-1^sec^-1^). We did, however, find that the inhibitory activity of α_1_-AT is greatly decreased in the presence of SP-A (*K*_ass _1.1 × 10^7 ^M^-1^sec^-1^, Figure 4), indicating that binding of SP-A to the carbohydrate moiety of α_1_-AT is not the only mechanism involved. The turnover number of the SP-A/deglycosylated α_1_-AT is 12, half that displayed by native α_1_-AT (Figure 4, 5). To explore other mechanisms of binding between SP-A and α_1_-AT, we incubated boiled SP-A and α_1_-AT. We found that boiled SP-A/native α_1_-AT displayed the same *K*_ass _and the same turnover number as native SP-A/deglycosylated α_1_-AT (Figures 4, 5). We postulated that SP-A carbohydrate chains could bind α_1_-AT, possibly through the amino acid backbone. In fact, carbohydrate chains isolated from SP-A mixed with deglycosylated α_1_-AT resulted in the same *K*_ass _and turnover number as those of native SP-A/deglycosylated α_1_-AT (Figures 4, 5). Besides these mechanisms of binding of SP-A to α_1_-AT, a third mechanism, i.e. a carbohydrate/carbohydrate interaction, probably exists since boiled SP-A and native α_1_-AT displayed a *K*_ass _of 1.9 × 10^7 ^M^-1^sec^-1 ^and ~6 turnovers (Figure 4, 5).

Finally, we studied the binding of deglycosylated SP-A to α_1_-AT. The *K*_ass _of native α_1_-AT mixed with deglycosylated SP-A was 1.2 × 10^7 ^M^-1^sec^-1 ^and the turnover number 18 (Figure 4, 5). Absence of SP-A/α_1_-AT binding, i.e. *K*_ass _3.4 × 10^7 ^M^-1^sec^-1^, and a turnover number of 1, was achieved by two combinations: 1) SP-A deglycosylated and boiled with native α_1_-AT, and 2) both proteins deglycosylated (Figures 4, 5). In the former case, absence of SP-A carbohydrates and denaturation of CRDs hindered any possible binding of SP-A to native α_1_-AT. In the latter case, the binding was precluded by the absence of carbohydrates on both proteins, in spite of the presence of intact CRDs in the SP-A.

## Discussion

The present data provide evidence for an *in vitro *interaction between SP-A and α_1_-AT. These glycoproteins belong to two systems of the lung that are supposed to act independently: the surfactant system and the proteinase/proteinase inhibitor system. Nevertheless, evidence for possible links between the two systems does exist. As an example, it has been shown that SP-A may be digested by elastolytic enzymes [28,29], and that inhalation of α_1_-AT in patients with cystic fibrosis may result in an increase of SP-A levels in bronchoalveolar lavage fluid (BALf) [30]. In addition, SP-D induces the production of matrix metalloproteinases by human alveolar macrophages [31], whereas the cysteine proteinase cathepsin H is involved in the first N-terminal processing step of SP-C [32]. The two systems may therefore interact in the lungs, both in physiologic and in pathologic pathways. The concentration of SP-A in the BALf of normal subjects is estimated to be ~277 nM [33]. Since approximately 1% of total SP-A is present in the free form [8,9], its concentration in BALf would be ~2.8 nM. Given that the concentration of α_1_-AT is ~5 μM [34], we reasoned that the two glycoproteins have a good chance of coming into contact during their life cycle.

Indeed our *in vitro *experiments indicate that the interaction between SP-A and α_1_-AT results in binding between them. This binding, which is calcium-dependent, appears to be complex since it could involve binding between the CRD of SP-A and carbohydrates on α_1_-AT, binding between SP-A carbohydrates and the protein backbone of α_1_-AT, and binding between the carbohydrate chains of both proteins.

Turnover per inactivation suggests that one part of SP-A binds 23 molar parts of α_1_-AT. Nevertheless, SP-A binds 11 molar parts of deglycosylated, fully active α_1_-AT (Figure 4, 5), thus suggesting a possible binding of SP-A carbohydrate chains to the amino acid backbone of α_1_-AT. Asn, to which carbohydrates of the native glycoprotein are linked [35], is a likely candidate. This hypothesis was confirmed by the results obtained with boiled SP-A and with isolated SP-A carbohydrate chains (Figure 4, 5). In support of this hypothesis, it has been reported that the binding of SP-A to influenza virus [36], herpes virus type 1 infected cells [37], and *M. tuberculosis *[38], involves N-linked carbohydrate chains on SP-A. Interestingly, there may be multiple binding sites on individual micro-organisms [12].

Our experiments also suggest a possible carbohydrate/carbohydrate interaction between SP-A and α_1_-AT. Such a type of linkage has been shown to operate in the calcium-mediated homotypic interaction between two Lewis (Le^x^) determinants (Galβ1→4[Fucα_1_→3]GlcNAc) involved in cell adhesion during murine embryogenesis [39]. Interestingly Le^x^-Le^x ^interactions appear to be calcium-dependent [40], by involving van der Waal forces. The fact that ultra-weak interactions are involved explains why this aspect is often underestimated [39-41].

It is difficult to postulate whether the three proposed mechanisms of binding take place simultaneously between native proteins. It may be that the CRD plays the main role and that the other two mechanisms are less important or take place only as artificial mechanisms once the proteins have been manipulated.

The binding with SP-A results in a decrease in the inhibition of HNE by α_1_-AT. There are several known mechanisms that could explain the inactivation of α_1_-AT. Beside the physiologic irreversible suicide substrate mechanism by which α_1_-AT inhibits HNE [42], α_1_-AT may also be inactivated by oxidation of methionine residue(s) located at or near the active site [22,23]. Another mechanism of α_1_-AT inactivation is proteolytic degradation at or near the active site by a number of host and non-host, mostly microbial, proteinases [42]. Whether these mechanisms may act *in vivo*, thereby contributing to the imbalance between proteinases and inhibitors in the pathogenesis and progression of pulmonary emphysema, is still a debated issue.

With respect to the inhibitory activity of α_1_-AT, that of Z α_1_-AT is further impaired by this latter's enhanced tendency to undergo spontaneous polymerization [2]. This phenomenon, also known as loop-sheet polymerization, likely accounts for why Z α_1_-AT is less efficient at inhibiting HNE, and has been demonstrated to be present *in vivo*, since Z α_1_-AT polymers have been detected in the BALf of Z α_1_-AT subjects with emphysema [45]. We found that SP-A binds Z α_1_-AT and that the binding further reduces the *K*_ass_, which is already impaired with respect to that of α_1_-AT. Were this binding to happen *in vivo*, it would further decrease the antiproteinase activity of Z α_1_-AT.

**Figure 6:**
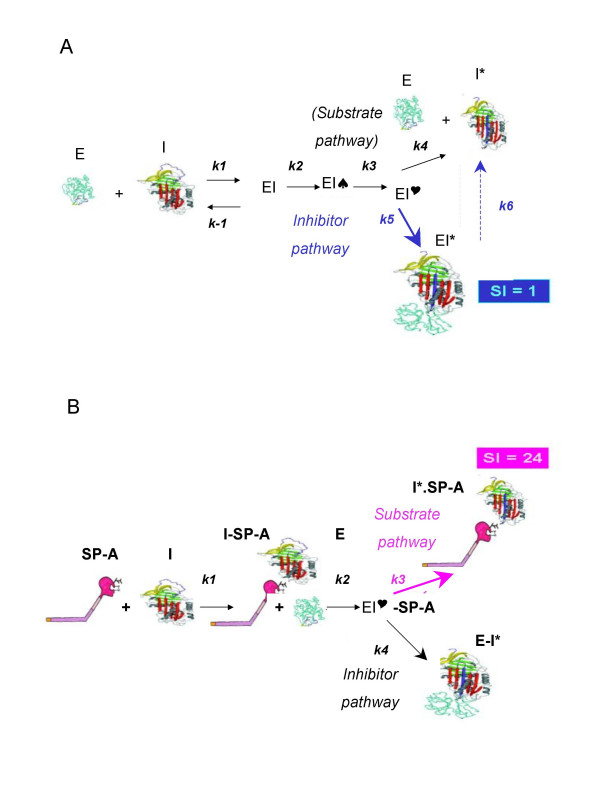
**Hypothetical mechanism of SP-A interference with α_1_-AT (simplification)**. **A**: interaction of α_1_-AT (I) with HNE (E). After an initial non-covalent Michaelis-like complex (EI), the interaction progresses through a tetrahedral intermediate (EI ♠), forming a covalent acyl-enzyme intermediate (EI ♥). The substrate pathway results in free HNE and cleaved α_1_-AT (I*); the inhibitory pathway results in a, about 100%, kinetically trapped loop-inserted covalent complex (E-I*). **B**: the SP-A (here shown as a trimer) interacts with α_1_-AT. In the presence of HNE, the formation of a covalent complex E-I* almost precluded (about 4%), and the reaction progresses through the substrate pathway towards free E and I* (cleaved α_1_-AT) – SP-A (96%). SI = stoichiometry of inhibition

The mechanism by which SP-A binding interferes with the α_1_-AT inhibitory mechanism is open to speculation. α_1_-AT inactivation taking place *in vitro *upon interaction between the two glycoproteins seems to occur because of the functional slowdown of α_1_-AT in the presence of SP-A, the turnover number shifting from 1 to 24. After an initial, non-covalent, Michaelis-like complex, the reaction between α_1_-AT and HNE progresses, through an acyl-enzyme intermediate resulting from peptide bond hydrolysis, to either a loop-inserted covalent complex (*inhibitory pathway*) or a cleaved serpin and free proteinase (*non-inhibitory or substrate pathway*) [42]. The number of turnovers for native α_1_-AT is 1 (Figure 5), indicating that the reaction inhibitor-HNE progresses towards the inhibitory pathway on the other side (Figure 6A). The number of turnovers after the incubation of native α_1_-AT or Z α_1_-AT with native SP-A is 24 (Figure 5), thus indicating that for α_1_-AT bound to SP-A the inhibitory pathway is precluded, and that the reaction inhibitor – HNE progresses mostly through the substrate pathway (Figure 6B).

In spite of the detailed dissection of the binding mechanism of SP-A to α_1_-AT *in vitro*, an obvious limitation of the present paper is the lack of specific studies investigating a possible interaction between SP-A and α_1_-AT *in vivo*. Nevertheless, some indirect evidence suggesting that such an interaction might take place is available, although it is not possible to address a plausible expectation of physiologic or pathophysiologic relevance of these findings. For example, a recent report has shown that in human sputum supramolecular complexes with heparan sulfate/Syndecan-1 and proteinase and inhibitors are present [46]. These complexes contain the proteinase inhibitors SLPI and α_1_-AT, NE as well, whose proteolytic activity is however not decreased . The large MW of SP-A makes difficult to highlight the occurrence of such supramolecular complexes including α_1_-AT by standard techniques [47]. Nevertheless, a report focusing on two-dimensional electrophoretic characteristics of BALf proteins in subjects affected by interstitial lung diseases [48] has intriguingly shown that some α_1_-AT fragments were superimposed on spots of SP-A, in its upper, acidic position. These findings, confirmed by mass spectrometric MALDI-TOF analysis, would suggest a possible SP-A/α_1_-AT interaction taking place *in vivo*.

## Conclusion

We have shown that SP-A binds α_1_-AT, and that this binding results in a significant decrease in the association rate constant of α_1_-AT for HNE. The mechanism of the binding seems to be predominantly mediated by the SP-A CRDs, as indicated by the calcium dependence and by the turnovers for inactivation, but other mechanisms may be involved, such as an interaction between SP-A carbohydrates and the α_1_-AT amino acid backbone or between carbohydrate chains of both glycoproteins. The presence of these complex binding mechanisms would exclude the hypothesis that the α_1_-AT inhibition occurred simply due to steric inhibition of the large SP-A molecule, but it would rather suggest a programmed, coordinated mechanism.

The *in vitro *interaction described here, if present *in vivo*, would be a novel mechanism of impairment of α_1_-AT inhibitory activity. It might represent a physiologic mechanism of regulating α_1_-AT activity, especially in acute conditions (for example during defense against infections agents) [49], in which an excess of α_1_-AT would interfere with the physiologic role of proteinases. α_1_-AT is indeed a highly specialised proteinase inhibitor [50], but the presence in nature of several, robust mechanisms of α_1_-AT downregulation (i.e. inherited deficiency, susceptibility to oxidative stress and proteolysis, polymerization) would imply the occurrence of intrinsic risks related to the overexpression of a nearly perfect and immortal inhibitor. Therefore, the formation of supramolecular complexes SP-A/α_1_-AT might be a sort of reserve mechanism, taking place in case of need.

On the other hand, the interaction with SP-A would be of particular relevance in the pathogenesis of pulmonary emphysema associated with α_1_-AT deficiency, since it would contribute significantly to the complex mechanisms of imbalance between Z α_1_-AT and HNE in the lungs. Obviously, all these speculations need further investigations, first of all to understand whether or not SP-A/α_1_-AT binding is a relevant down-regulatory mechanism of α_1_-AT inhibitory activity *in vivo*.

## Abbreviations

α1-AT, α1-antitrypsin

αChy, α chymotrypsin

(CH_3_)_2_SO, dimethylsulfoxide

CRD, carbohydrate recognition domain

CRF, collagenase-resistant fragment

HNE, human neutrophil elastase

MNOCH_3, _methyl-α-D-mannopyranoside

PNA, p-nitroanilide

SP-A, surfactant protein-A

## Competing interests

The author(s) declare that they have no competing interests.

## Authors' contributions

MG participated in the study design, performed most experiments, and helped to draft the manuscript. AL participated in the deglycosylation experiments and carbohydrate chains isolation. PI designed the experiments for the α1-AT/SP-A complex identification, and helped to draft the manuscript. CDS participated in the kinetic studies. PR performed the experiments for the α1-AT/SP-A complex identification. DD performed the purification of SP-A and CRF. NC took part to some kinetic experiments. EP participated in the coordination of the study. AB performed the purification of SP-A and CRF, helped to draft the manuscript and critically reviewed it. ML conceived the study, participated in its design, and coordinated the manuscript final version. All authors read and approved the final manuscript.

## Supplementary Material

Additional File 1 contain portion of Methods' section and include details on modification of native proteins used in the experiments and details of kinetic procedures. Click here for file

## References

[B1] Travis J, Salvesen GS (1983). Human plasma proteinase inhibitors. Annu Rev Biochem.

[B2] Carrell RW, Lomas DA (2002). Alpha_1_-antitrypsin deficiency. A model for conformational diseases. N Engl J Med.

[B3] Sastry K, Ezekowitz RA (1993). Collectins: pattern recognition molecules involved in the first line of host defense. Curr Opin Immunol.

[B4] Haagsman HP, White RT, Schilling J, Lau K, Benson BJ, Golden J, Hawgood S, Clements JA (1989). Studies of the structure of lung surfactant protein SP-A. Am J Physiol.

[B5] McCormack FX, Whitsett JA (2002). The pulmonary collectins, SP-A and SP-D, orchestrate innate immunity in the lung. J Clin Invest.

[B6] Bhattacharyya SN, Lynn WS (1977). Structural studies and oligosaccharides of glycoprotein isolated from alveoli of patients with alveolar proteinosis. J Biol Chem.

[B7] Munakata H, Nimberg RB, Snider GL, Robins AG, Van Halbeek H, Vliegenthart JF, Schmid K (1982). The structure of the carbohydrate units of the 36K glycoprotein derived from the lavage of a patient with alveolar proteinosis by high-resolution ^1^H-NMR spectroscope. Biochem Biophys Res Commun.

[B8] Baritussio A, Alberti A, Quaglino D, Pettenazzo A, Dal Zoppo D, Sartori L, Pasquali-Ronchetti I (1994). SP-A, SP-B, and SP-C in surfactant subtypes around birth: reexamination of alveolar life cycle of surfactant. Am J Physiol.

[B9] Savov J, Wright JR, Young SL (2000). Incorporation of biotinylated SP-A into rat lung surfactant layer, type II cells, and Clara cells. Am J Physiol.

[B10] Wright JR (1997). Immunomodulatory functions of surfactant. Physiol Rev.

[B11] Korfhagen TR (2001). Surfactant protein A (SP-A)-mediated bacterial clearance. SP-A and cystic fibrosis. Am J Respir Cell Mol Biol.

[B12] Mason RJ, Greene K, Voelker DR (1998). Surfactant protein A and surfactent protein D in health and disease. Am J Physiol.

[B13] Mega T, Lujan E, Yoshida A (1980). Studies on the oligosaccharide chains of human α1-proteinase inhibitor. I. Isolation of glycopeptides. J Biol Chem.

[B14] Lupi A, Viglio S, Luisetti M, Gorrini M, Coni P, Faa G, Cetta G, Iadarola P (2000). α1-antitrypsin in serum determined by capillary isoelectric focusing. Electrophoresis.

[B15] Ferrarotti I, Baccheschi J, Zorzetto M, Tinelli C, Corda L, Balbi B, Campo I, Pozzi E, Faa G, Coni P, Massi G, Stell G, Luisetti M (2005). Prevalence and phenotype of subjects carrying rare variants in the Italian Registry for alpha1-antitrypsin deficiency. J Med Genet.

[B16] Howgood S, Benson BJ, Shilling J, Damm D, Clements JA, White RT (1987). Nucleotide and amino acid sequence of pulmonary surfactant protein SP18 and evidence for co-operation between SP18 and SP28-36 in surfactant lipid adsorption. Pro Natl Acad Sci USA.

[B17] Alberti A, Luisetti M, Braschi A, Rodi G, Iotti G, Sella D, Poletti V, Benori V, Baritussio A (1996). Broncho-alveolar lavage fluid composition in alveolar proteinosis. Early changes after therapeutic lavage. Am J Respir Crit Care Med.

[B18] Wright JR, Zlogar DF, Taylor JC, Zlogar TM, Restepo CI (1999). Effect of endotoxin on surfactant protein A and D stimulation of NO production by alveolar macrophages. Am J Physiol.

[B19] Meloni F, Alberti A, Bulgheroni A, Lupi A, Paschetto E, Marone Bianco A, Rodi G, Fietta A, Luisetti M, Baritussio A (2002). Surfactant apoprotein A modulates interleukin-8 and monocyte chemotactic peptide-1 production. Eur Respir J.

[B20] Vincent J-P, Lazdunski M (1972). Trypsin-pancreatic trypsin inhibitor associations. Dynamics of the interaction and role of disulfide bridges. Biochemistry.

[B21] Beatty K, Bieth J, Travis J (1980). Kinetics of association of serine proteinases with native and oxidized α-1-proteinase inhibitor and α-1-antichymotrypsin. J Biol Chem.

[B22] Gorrini M, Lupi A, Viglio S, Pamparana F, Cetta G, Iadarola P, Powers JC, Luisetti M (2001). Inhibition of human neutrophil elastase by erythromycin and flurythromycin, two macrolide antibiotics. Am J Respir Cell Mol Biol.

[B23] Creuwels LAJM, van Golde LMG, Haagsman H (1997). The pulmonary surfactant system: biochemical and clinical aspects. Lung.

[B24] Ogushi F, Fells GA, Hubbard RC, Straus SD, Crystal RG (1987). Z-type α1-antitrypsin is less competent than M1-type α1-antitrypsin as an inhibitor of neutrophil elastase. J Clin Invest.

[B25] Haagsman HP, Sargeant T, Hauschka VH, Benson BJ, Hawgood S (1990). Binding of calcium to SP-A, a surfactant-associated protein. Biochemistry.

[B26] Haurum JS, Thiel S, Haagsman HP, Laursen SB, Larsen B, Jensenius JC (1993). Studies on the carbohydrate-binding characteristics of human pulmonary surfactant-associated protein A and comparison with two other collectins: mannan-binding protein and conglutinin. Biochem J.

[B27] Khubchandani KR, Oberley RE, Snyder JM (2001). Effects of surfactant protein A and NaCl concentration on the uptake of *Pseudomonas aeruginosa *by THP-1 cells. Am J Respir Cell Mol Biol.

[B28] Rubio F, Cooley J, Accurso FJ, Remold-O'Donnell E (2004). Linkage of neutrophil serine proteases and decreased surfactant protein-A (SP-A) levels in inflammatory lung disease. Thorax.

[B29] Beatty AL, Malloy JL, Wright JR (2005). *Pseudomonas aeruginosa *degrades pulmonary surfactant and increases conversion *in vitro*. Am J Respir Cell Mol Biol.

[B30] Griese M, von Bredow C, Birrer P (2001). Reduced proteolysis of surfactant protein A and changes of the bronchoalveolar lavage fluid proteome by inhaled alpha 1-protease inhibitor in cystic fibrosis. Electrophoresis.

[B31] Crippes Trask B, Malone MJ, Lum EH, Welgus HG, Crouch EC, Shapiro SD (2001). Induction of macrophage matrix metalloproteinase biosynthesis by surfactant protein D. J Biol Chem.

[B32] Brasch F, ten Brinke A, Johnen G, Ochs M, Kapp N, Müller KM, Beers MF, Fehrenbach H, Richter J, Batenburg JJ, Bühling F (2002). Involvement of cathepsin H in the processing of the hydrophobic surfactant-associated protein C in type II pneumocytes. Am J Respir Cell Mol Biol.

[B33] Van de Graaf EA, Jansen HM, Lutter R, Alberts C, Kobsen J, de Vries IJ, Out TA (1992). Surfactant protein A in bronchoalveolar lavage fluid. J Lab Clin Med.

[B34] Rennard SI, Ghafouri M, Thompson AB, Linder J, Vaughan W, Jones K, Ertl RF, Christensen K, Prine A, Stahl MG (1990). Fractional processing of sequential bronchoalveolar lavage to separate bronchial and alveolar samples. Am Rev Respir Dis.

[B35] Sprio RG (2000). Protein glycosylation: nature, distribution, enzymatic formation, and disease implications of glycopeptide bonds. Glycobiology.

[B36] Benne CA, Kraaijeveld CA, Van Strijp JAG, Brouwer E, Harmsen M, Verhoef J, van Golde LMG, van Iwaarden JF (1995). Interactions of surfactant protein A with influenza A viruses: binding and neutralization. J Infect Dis.

[B37] Van Iwaarden JF, Van Strijp JAG, Visser H, Haagsman HP, Verhoef J, van Golde LMG (1992). Binding of surfactant protein A (SP-A) to herpes simplex virus type 1-infected cells is mediated by the carbohydrate moiety of SP-A. J Biol Chem.

[B38] Gaynor CD, McCormack FX, Voelker DR, McGowan SE, Schlesinger LS (1995). Pulmonary surfactant protein A mediates enhanced phagocytosis of *Mycobacterium tuberculosis *by a direct interaction with human macrophages. J Immunol.

[B39] Pincet F, Le Bouar T, Zhang Y, Esnault J, Mallet J-M, Perez E, Sinaÿ P (2001). Ultraweak sugar-sugar interactions for transient cell adhesion. Biophys J.

[B40] Henry B, Desvaux H, Pritstchepa M, Berthault P, Zhang Y, Mallet J-M, Esnault J, Sinaÿ P (1999). NMR study of a Lewis^x ^pentasaccharide derivative: solution structure and interaction with cations. Carbohydr Res.

[B41] Sears P, Wong C-H (1996). Intervention of carbohydrate recognition by proteins and nucleic acids. Proc Natl Acad Sci USA.

[B42] Silverman GA, Bird PI, Carrell RW, Church FC, Coughlin PB, Gettings PGW, Irving JA, Lomas DA, Luke CJ, Moyer RW, Pemberton PA, Remold-O'Donnell E, Salvesen GS, Travis J, Whisstock JC (2001). The serpins are an expanding superfamily of structurally similar but functionally diverse proteins. J Biol Chem.

[B43] Taggart C, Cervantes-Laurean D, Kim G, McElvaney NG, Wehr N, Moss J, Levine RL (2000). Oxidation of either methionine 351 or methionine 358 in α1-antitrypsin causes loss of anti-neutrophil elastase activity. J Biol Chem.

[B44] Travis J, Shieh B-H, Potempa J (1988). The functional role af acute phase plasma proteinase inhibitors. Tokai J Exp Clin Med.

[B45] Elliott P, Bilton D, Lomas DA (1998). Lung polymers in Z α_1_-antitrypsin related emphysema. Am J Respir Cell Mol Biol.

[B46] Chan SCH, Shum DKY, Ip MSM (2003). Sputum sol neutrophil elastase activity in bronchiectasis. Differential modulation by Syndecan-1. Am J Respir Crit Care Med.

[B47] Madsen J, Kliem A, Nielsen O, Koch C, Steinhilber W, Holmskov U (2003). Expression and localization of lung surfactant protein A in human tissues. Am J Respir Cell Mol Biol.

[B48] Magi B, Bini L, Perari MG, Fossi A, Sanchez JC, Hochstrasser D, Paesano S, Raggiaschi R, Santucci A, Pallini V, Rottoli P (2002). Bronchoalveolar lavage fluid protein composition in patients with sarcoidosis and idiopathic pulmonary fibrosis: a two-dimensional electrophoretic study. Electrophoresis.

[B49] Matthay MA, Zimmerman GA (2005). Centennial Review. Acute lung injury and the acute respiratory distress syndrome. Four decades of Inquiry into pathogenesis and rational management. Am J Respir Cell Mol Biol.

[B50] Otlewski J, Jelen F, Zakreweska M, Oleksy A (2005). The many faces of protease-protein inhibitor interaction. EMBO J.

