# Supplementary material for: SP-A binds alpha1-antitrypsin in vitro and reduces the association rate constant for neutrophil elastase
Source: Respir Res. 2005 Dec 13;6(1):146. doi: 10.1186/1465-9921-6-146 (PMC1343571; doi:10.1186/1465-9921-6-146)
Supplement: Additional File 1 — contain portion of Methods' section and include details on modification of native proteins used in the experiments and details of kinetic procedures. [file 1465-9921-6-146-S1.doc]

SP-A Binds Alpha_1_-antitrypsin *In Vitro* and Reduces the Association Rate Constant for Neutrophil Elastase

Marina Gorrini, Anna Lupi, Paolo Iadarola, Conceição Dos Santos, Paola Rognoni, Daniele Dalzoppo, Natalia Carrabino, Ernesto Pozzi, Aldo Baritussio, and Maurizio Luisetti

# Additional Data Files

# Methods

*Modification of the native proteins*

A) A collagenase-resistant fragment (CRF) of SP-A was generated using a previuosly described procedure [1] with minor modifications. Briefly, 0.25 mg of SP-A in 50 mM Tris, 2 mM CaCl_2_, pH 7.4, were incubated at 37°C for 24 hrs in the presence of collagenase type III. At the end, digested SP-A was suspended in 5 mM Tris, 100 mM NaCl, 25 mM CaCl_2_, pH 7.4 (binding buffer) by buffer exchange and was then applied to a 3 ml column of D-mannose sepharose 4B pre-equilibrated with the same buffer. After 10 volumes lavages, digested SP-A was eluted with 5 volumes of 5 mM Tris, 100 mM NaCl, 2 mM EDTA, pH 7.4, and 2 μg were analyzed under reducing conditions by tricine-SDS-PAGE (10% T, 3% C), followed by Coomassie staining.

B) Boiled SP-A was prepared by boiling SP-A for 10 min at 100°C.

C) Deglycosylated SP-A and α_1_-AT were obtained by dissolving them in 50 mM phosphate buffer pH 7.5, and then adding N-glycosidase F (final concentration 0.06 mg/ml) [2]. The reaction was carried out at 37°C for 5 days for SP-A and for 3 days for α_1_-AT. The proteins were then extensively dialyzed at 4°C against 10 mM phosphate buffer pH 7.4 (MW cut-off 12,000-14,000) and concentrated with Microcon concentrators (MW cut-off 10,000) (Amicon). To test the effectiveness of deglycosylation, proteins were analyzed by SDS-PAGE (5% stacking gel, 12% running gel) followed by Coomassie staining.

D) Isolation of the polysaccharide chains of SP-A**.** One hundred micrograms of SP-A, in 50 mM phosphate buffer pH 7.5 with 2% SDS and 0.7 M β-mercaptoethanol, were heated at 100°C for 5 minutes, and then exposed to N-glycosidase F (MW 36,000) for 3 hours at 37°C in the presence of Triton X 100 (final concentration 15% v/v). The liberated polysaccharide chains were recovered by centrifuging the reaction mixture in Microcon concentrators (MW cut-off: 10,000). Carbohydrates were measured in the ultrafiltrate and in the retentate with the Molish test, using a standard curve of glucose ranging from 0 to 1,000 μg/ml. Briefly, standards and samples (0.1 ml) were mixed with 0.1 ml of 5% phenol in water and 0.5 ml of concentrated sulfuric acid and were then left to stand for 10 minutes. Tubes were then incubated at 25°C for 15 minutes and the absorbance read at 488 nm against distilled water.

*Kinetics studies*

**1)** Active site titrations of α_1_-ATs and coupled α_1_-ATs/SP-A were performed by incubating 7.5 nM of αChy (previously titrated as 99.7% active with TosPheCMK) for 15 min with inhibitors at 25°C and then free αChy activity was evaluated with SucAlaAlaProPheNA.

**2)** Reaction order and K_ass_ for αChy were determined by incubating 7.5 nM αChy with α_1_-ATs and coupled α_1_-ATs/SP-A (at concentrations corresponding to 100% inhibition) for 15 min at 25°C. αChy activity was measured at 5, 10, 15, 30, and 60 min, as previously described.

**3)** K_ass_ for HNE was evaluated by incubating α_1_-ATs and α_1_-ATs/SP-A complex with αChy (from 7.5 to 100 nM) and HNE (7.5 nM) for 15 min at 25°C. HNE activity was measured.

**4)** Turnover numbers per inactivation were derived from a series of experiments: mixtures containing α_1_-ATs and α_1_-ATs/SP-A complex (SP-A 20 nM and α_1_-ATs from 0 to 600 nM) and HNE in molar ratios from 0 to 30, were prepared and let to stand for 15 min at 37°C. Samples were then filtered with Microcon Y-M 100 (MW cut-off 100,000; Amicon, Italy) 10,000 rpm for 12 min at 25°C. Upper solutions (SP-A/α_1_-ATs complexes) and lower solutions (free α_1_-ATs) were recovered, and assayed for total protein content (Quick Start Bradford Protein Assay; Bio-Rad Laboratories, Hercules CA, USA), then 20 nM HNE was added and the solutions kept for 16 hrs at 37°C (for α_1_-AT) or 24 hrs at 25°C to decrease spontaneous polymerization of Z α_1_-AT . The residual HNE activity was then determined.

# References

1. Haagsman HP, Hagwood S, Sargeant T, Buckley D, White RT, Drickamer K, Benson BJ : **The major lung surfactant protein, SP 28-36, is a calcium-dependent, carbohydrate-binding protein.** *J Biol Chem* 1987, **262**: 13877 – 13880.
2. Tarentino AL, Plummer TD Jr: **Enzymatic deglycosylation of asparagine-linked glycans: purification, properties, and specificity of oligosaccharide-cleaving enzymes from *Flavobacterium meningosepticum*.** *Methods Enzymol* 1994, **230**: 44 – 57.
